# Computational identification of a new SelD-like family that may participate in sulfur metabolism in hyperthermophilic sulfur-reducing archaea

**DOI:** 10.1186/1471-2164-15-908

**Published:** 2014-10-17

**Authors:** Gao-Peng Li, Liang Jiang, Jia-Zuan Ni, Qiong Liu, Yan Zhang

**Affiliations:** Key Laboratory of Nutrition and Metabolism, Institute for Nutritional Sciences, Shanghai Institutes for Biological Sciences, Chinese Academy of Sciences, University of Chinese Academy of Sciences, Shanghai, 200031 P. R. China; Key Laboratory of Systems Biology, Shanghai Institutes for Biological Sciences, Chinese Academy of Sciences, University of Chinese Academy of Sciences, Shanghai, 200031 P. R. China; College of Life Sciences, Shenzhen University, Shenzhen, 518060 Guangdong Province P. R. China

**Keywords:** Selenium, Sulfur, Selenophosphate synthetase, Bioinformatics, Comparative genomics, Archaea

## Abstract

**Background:**

Selenium (Se) and sulfur (S) are closely related elements that exhibit similar chemical properties. Some genes related to S metabolism are also involved in Se utilization in many organisms. However, the evolutionary relationship between the two utilization traits is unclear.

**Results:**

In this study, we conducted a comparative analysis of the selenophosphate synthetase (SelD) family, a key protein for all known Se utilization traits, in all sequenced archaea. Our search showed a very limited distribution of SelD and Se utilization in this kingdom. Interestingly, a SelD-like protein was detected in two orders of Crenarchaeota: Sulfolobales and Thermoproteales. Sequence and phylogenetic analyses revealed that SelD-like protein contains the same domain and conserved functional residues as those of SelD, and might be involved in S metabolism in these S-reducing organisms. Further genome-wide analysis of patterns of gene occurrence in different thermoproteales suggested that several genes, including SirA-like, Prx-like and adenylylsulfate reductase, were strongly related to SelD-like gene. Based on these findings, we proposed a simple model wherein SelD-like may play an important role in the biosynthesis of certain thiophosphate compound.

**Conclusions:**

Our data suggest novel genes involved in S metabolism in hyperthermophilic S-reducing archaea, and may provide a new window for understanding the complex relationship between Se and S metabolism in archaea.

**Electronic supplementary material:**

The online version of this article (doi:10.1186/1471-2164-15-908) contains supplementary material, which is available to authorized users.

## Background

Selenium (Se) is an important micronutrient in all three domains of life [1]. The major biological form of this trace element is selenocysteine (Sec), which is a Se analogue of cysteine (Cys) and incorporated into selenoproteins at an in-frame UGA codon [2–4]. All Sec-utilizing organisms use an elaborate synthetic and translational machinery for Sec biosynthesis and its insertion into a selenoprotein, which is different from the enzymatic system for the 20 canonical amino acids. Both common key components and differences exist among Sec-decoding mechanisms employed by archaea, bacteria and eukaryotes [3–8]. The mechanism of Sec biosynthesis in bacteria is best described in *Escherichia coli* [6, 8–10]. Several proteins and a specific tRNA are known to be involved in this process, including bacterial Sec synthase (SelA), Sec-specific elongation factor (SelB), Sec-tRNA^[Ser]Sec^ (SelC) and selenophosphate synthetase (SelD). In archaea and eukaryotes, substitute/additional components are needed for Sec biosynthesis, such as O-phosphoseryl-tRNA^[Ser]Sec^ kinase (PSTK), archaeal/eukaryotic Sec synthase (SecS), SECIS-binding protein 2 (SBP2) and ribosomal protein L30 [7, 11, 12]. In addition, Se is also found in 5-methylaminomethyl-2-selenouridine (SeU) which is located at the wobble position of the anticodons of some tRNAs, as well as in a Se-containing cofactor bound to certain moly-bdenum-containing hydroxylases [13–16]. Specific genes are known to be involved in each of these processes (SelD/YbbB for SeU trait [17] and SelD/YqeB/YqeC for Se-containing cofactor [16]).

Among known Se-related genes, SelD is considered as a key gene which is essential for all known Se utilization traits. It is a member of a small ATP-binding superfamily that also includes the purine biosynthetic enzymes PurM (phosphoribosylaminoimidazole synthetase) and PurL (formylglycinamide ribonucleotide amidotransferase), thiamine-monophosphate kinase (ThiL) and nickel-iron hydrogenase maturation protein HypE [18]. The function of SelD is to catalyze the activation of selenide with adenosine 5′-triphosphate (ATP) to generate selenophosphate, the essential Se donor for the formation of Sec and SeU residues in proteins and RNAs, respectively [19]. Several comparative genomics studies have shown that SelD gene could be used as a general signature for Se utilization in biology [16, 17, 20]. Since Sec is the main form of Se utilization, the majority of studies on SelD focused on its function and regulation during the process of Sec biosynthesis in a variety of model organisms [21–23]. In contrast, the evolutionary and functional relationships between SelD and other homologs are not fully understood.

On the other hand, Se and sulfur (S) are two closely related elements utilized for a vast array of biochemical reactions [24]. It has been known that some genes might be involved in the metabolism of both elements. For example, sulfate transporters are responsible for the uptake of selenate in bacteria [25]. It is also known that Sec lyases (SCLs) and Cys desulfurases (CDs) catalyze the removal of Se or S from Sec or Cys and generally act on both substrates, in spite that SCLs from higher eukaryotes (including human) are specific for Sec and do not accept Cys as substrate [24]. Thus, it would be interesting to investigate the relationship between Se and S metabolic pathways in different organisms.

In this study, we carried out a comparative and phylogenetic analysis of SelD family in archaea based on the rapidly increased genomes sequenced in this kingdom. Occurrence of SelDs and their homologs were identified, which revealed a limited utilization of Se in archaea. In addition, we found a new SelD-like family in several hyperthermophilic S-utilizing archaea, which contains the same catalytic Cys residue as those in SelD proteins. Phylogenetic and genome context analyses of SelD-like genes revealed that they are more likely to share a common ancestor with SelD genes and may participate in S metabolism, probably involved in the biosynthesis of certain thiophosphate compound in these organisms. Further bioinformatics approaches were used to analyze patterns of gene occurrence, and several genes that may co-occur with SelD-like gene were identified, implying functional links among them. Thus, our results provide new insights into the complex and dynamic relationship between Se and S metabolism in S-utilizing archaea.

## Results and discussion

### Distribution of SelD and Se utilization traits in archaea

In the recent decade, comparative genomics has become an important strategy for understanding the evolutionary dynamics of a variety of cellular pathways in different organisms. Several comparative studies on Se have been carried out to examine Se utilization traits and identify new genes involved in Se metabolism in prokaryotes and eukaryotes [16, 26–28]. For example, based on a comparative genomics analysis of SelD genes in bacteria, we successfully identified a new Se utilization trait as well as several genes involved in this pathway [16].

**Figure 1:**
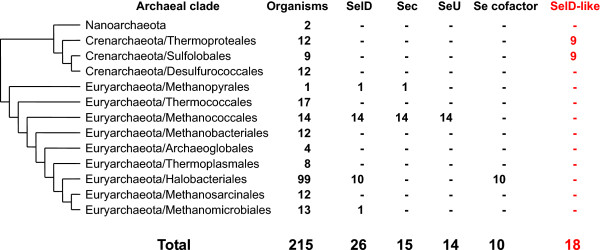
**Overview of distribution of SelD proteins and Se utilization traits in archaea.** The tree is based on 16S rRNA sequences, which was simplified to only show major branches. The numbers of organisms that contain SelD and known Se utilization traits are shown. Distribution of organisms containing SelD-like family is highlighted in red.

In this study, we analyzed the distribution of both SelD gene and the three known Se utilization pathways in different archaeal taxa (Figure 1; details are shown in Additional file 1: Table S1). Among all sequenced organisms, only 26 (12.1%) contain SelD genes. This is consistent with our previous observation that Se utilization is quite restricted in this kingdom of life [28]. Further investigation of the three known Se utilization traits revealed that 15 Sec-utilizing (SelD + SelA + SelB), 14 SeU-utilizing (SelD + YbbB) and 10 Se-cofactor-utilizing (SelD + YqeB + YqeC) organisms were present in different clades. The SeU and Se-containing cofactor utilization traits were only found in Methanococcales and Halobacteriales, respectively. Only methanococcales have both Sec-decoding and SeU-utilizing traits.

Surprisingly, a newly sequenced archaeon, *Methanoplanus petrolearius* (belonging to the order Euryarchaeota/Methanomicrobiales), was found to have an orphan SelD gene and lack components of any known Se utilization trait. The SelD protein in this organism is a typical SelD which is highly similar to other orthologs (say, 51% sequence identity when compared with *Methanocaldococcus jannaschii* SelD using BLAST) and contains a conserved Cys residue in the active site. In a separate study, orphan SelD genes were also found in some newly sequenced bacteria, implying an additional but unknown SelD-dependent use of Se (unpublished data).

### Identification of a new SelD-like family in crenarchaeota

**Figure 2:**
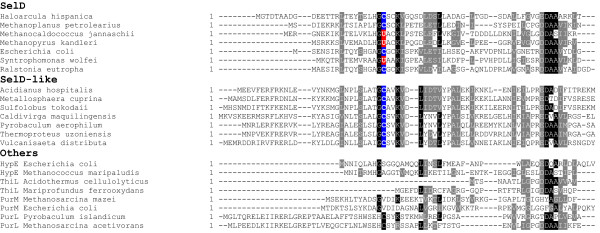
**Multiple sequence alignments of SelD and SelD-like proteins.** The alignments show the N terminal regions that contain the active site in representative SelD and SelD-like proteins. Conserved residues are highlighted. Sec (U) and the corresponding Cys (C) residues are shown in red and blue, respectively. The conserved Cys residue corresponding to the catalytic Sec/Cys in the active site of SelD could not be detected in other families of the ATP-binding superfamily that contains SelD.

Several distant homologs of SelD (named SelD-like thereafter) were exclusively found in two orders of the phylum Crenarchaeota: Sulfolobales and Thermoproteales, in which all sequenced organisms do not have any known Se utilization trait (Figure 1 and Additional file 1: Table S1). The sequence similarities between SelD and SelD-like proteins are low (for example, only 26% identity was observed between *M. jannaschii* SelD and *Sulfolobus acidocaldarius* SelD-like). However, these SelD-like proteins contain the same domain as SelD (COG0709) and the conserved Cys residue corresponding to the catalytic Sec/Cys in the active site of SelD, both of which are not detected in other families of the ATP-binding superfamily that contains SelD (Figure 2 and Additional file 2: Figure S1; SelD-like and SelD sequences are available in Additional file 3). Further analysis of the occurrence of SelD-like protein in bacteria and eukaryotes revealed that it is absent in all sequenced organisms in the two kingdoms.

Recently, the crystal structures of SelD have been solved in several bacteria, which provide important basis for understanding the catalytic mechanism of SelD-mediated selenophosphate synthesis. For example, based on the crystal structures of *Aquifex aeolicus* SelD and its complex with ATP analogue, Itoh *et al.* found that four aspartic acid (Asp) residues may coordinate metal ions (cobalt or magnesium) to bind the phosphate groups of ATP [29]. In addition, the Sec/Cys-X-X-Lys motif is also essential for SelD activity. Very recently, the crystal structure of a C17S mutant of *E. coli* SelD also confirmed that these Asp residues which are involved in the binding of magnesium at the active site to allow the interaction with ATP, and a conserved asparagine (Asn87 in *E. coli* SelD), play an essential role for catalysis in *E. coli* SelD [30]. Interestingly, all of these important residues are present in all SelD-like proteins (at either the same or very close position based on sequence alignment analysis, Additional file 2: Figure S1). Thus, the SelD-like family may also bind certain metal (e.g., magnesium) and use a similar mechanism for the catalytic reaction of its substrate (most likely ATP or its analogue).

**Figure 3:**
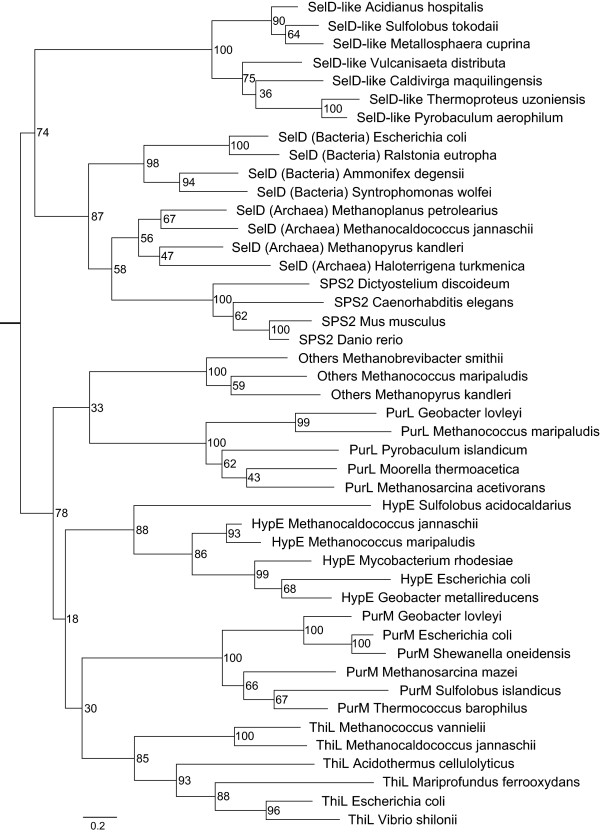
**Phylogenetic analysis of the ATP-binding superfamily that contains SelD and other families.** The tree is built based on representative sequences of each family. Bootstrapping values are included. The measurement of distance for the branch length is indicated.

Considering that SelD belongs to an ATP-binding superfamily which also includes PurM, PurL, ThiL and HypE families, we collected proteins from representative organisms for each of them. Phylogenetic analysis revealed that SelD-like proteins are different from SelD family and form a separate branch of this superfamily (Figure 3). The evolutionary relationship between SelD and SelD-like proteins appeared to be closer than other families, implying that SelD-like and SelD proteins have a common ancestor. Considering that all sequenced crenarchaeota lack known Se utilization trait, we assume that these SelD-like proteins may be unrelated to Se metabolism although their function is unclear so far. However, the possibility that SelD-like protein may participate in an unknown Se-related process in these organisms could not be completely excluded.

### Functional analysis of SelD-like proteins

**Figure 4:**
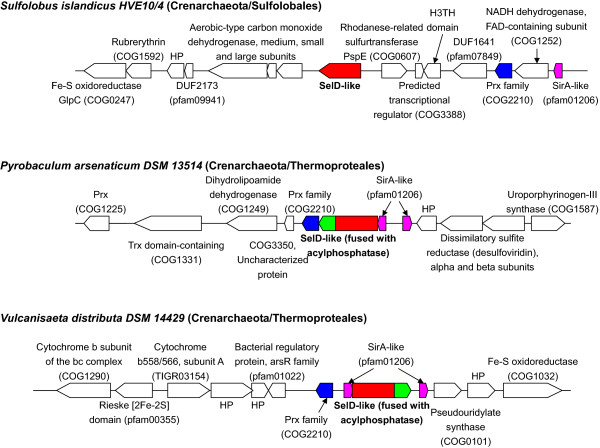
**Genomic context of SelD-like gene in representative genomes.** Functionally related genes in the genomes of *Sulfolobus islandicus HVE10/4*, *Pyrobaculum arsenaticum DSM 13514* and *Vulcanisaeta distributa DSM 14429* are color coded. Coding direction is also indicated. HP represents hypothetical proteins.

As mentioned above, genes encoding SelD-like proteins were only detected in Sulfolobales and Thermoproteales; however, Desulfurococcales, the third order of Crenarchaeota, lack this gene. This may suggest that SelD-like protein evolved in the ancestor of crenarchaeota and lost in desulfurococcales. To further study the possible function of SelD-like proteins, we examined genes that are located within 10,000 nucleotides upstream and downstream of SelD-like gene in all organisms containing this gene. It has been known for some time that many clustered genes, especially those organized in one operon, may be functionally related [31]. Thus, it is possible to identify functional partners of SelD-like gene based on gene neighborhood analysis. The genomic context of SelD-like gene in representative genomes is shown in Figure 4. A comprehensive analysis of genomic context of SelD-like gene in all completely sequenced sulfolobales and thermoproteales genomes is shown in Additional file 2: Figure S2.

First, all SelD-like proteins in Thermoproteales were found to be fused with an acylphosphatase-like domain. Acylphosphatase belongs to a large protein family (COG1254) that specifically catalyzes the hydrolysis of the carboxyl-phosphate bond of acylphosphate substrates such as carbamoylphosphate and 1,3-biphosphoglycerate, and results in the production of carboxylate and phosphate [32]. It is widely distributed in all three domains of life (including all sulfolobales) and has been found to be involved in a number of physiological processes such as the regulation of the glycolytic pathway and pyrimidine biosynthesis, though its biological function is as yet unknown [33]. Several recent structure-based studies of acylphosphatase from different hyperthermophilic archaea such as *S. solfataricus* and *Pyrococcus horikoshii* have revealed that normally folded acylphosphatases retain a significant tendency to form amyloid fibrils through a direct assembly of monomers in their native-like topology [34, 35]. The fusion of SelD-like and acylphosphatase-like domains indicates that there might be a strong functional link between acylphosphatase-like and SelD-like proteins. It is possible that this acylphosphatase-like domain is involved in hydrolysis of certain acylphosphate-like substrate that is synthesized by SelD-like protein. It is also possible, but less likely, that acylphosphate-like protein may provide phosphate substrate for the proper function of SelD-like protein (instead of ATP).

A second interesting finding is that several common genes were found to be localized very close to SelD-like genes in both sulfolobales and thermoproteales, including genes encoding SirA-like (pfam01206) and peroxiredoxin(Prx)-like (COG2210) proteins. The SirA-like protein is considered as a S transfer protein that belongs to a predicted redox protein superfamily and has been found to be associated with SelD [16]. Homologs of SirA-like proteins are widespread in almost all prokaryotes and may have different functions. The Prx-like protein is a distant homolog of Prx family, which is a ubiquitous family of antioxidant enzymes and shares the same basic catalytic mechanism, in which a redox-active Cys in the active site is oxidized to a sulfenic acid by the peroxide substrate [36]. Our gene neighborhood analysis suggested that both redox proteins may participate in the cellular processes involving SelD-like proteins in archaea.

Although there is no clear evidence with regard to the function of SelD-like, we could always find that genes associated with S metabolic pathways are located nearby. Besides SirA-like and Prx-like, additional S-related genes were also observed in almost all sequenced crenarchaeota containing SelD-like, such as rhodanese-related sulfurtransferase PspE, dissimilatory sulfite reductase (DSR) and Fe-S oxidoreductases (Figure 4 and Additional file 2: Figure S2). Thus, it is possible that SelD-like protein is involved in S metabolism. Considering that SelD catalyzes the biosynthesis of selenophosphate from selenide and ATP, plus that SelD-like proteins have the same functional residues as SelD, we finally hypothesized that SelD-like protein might be involved in biosynthesis of certain thiophosphate molecule using S-containing molecule (sulfide?) and ATP/ATP analogue. The product of SelD-like might be similar to selenophosphate where the Se atom is replaced with S to form a P=S double bond (named thiophosphate (P=S) thereafter). This is different from many other thiophosphate compounds containing S-P single bond(s). However, the restricted distribution of SelD-like gene implied that it might be only needed for S utilization in sulfolobales and thermoproteales, all of which are hyperthermophilic S-reducing archaea. Instead, more and more desulfurococcales species are known to be unable to use S or S-containing compounds for energy formation, which may probably result in the loss of this gene in the Desulfurococcales order of Crenarchaeota. Further experimental studies are needed to validate the biological function of SelD-like protein and the presence and/or function of such thiophosphate compound.

### Computational search for additional genes associated with SelD-like gene

SelD-like gene could be detected in all sulfolobales and almost all thermoproteales. However, it was absent in three completely sequenced thermoproteales including *Pyrobaculum islandicum*, *P. neutrophilum* and *Thermofilum pendens*, implying a very recent loss of this gene in these organisms. Based on the comparison of genomic sequences of both SelD-like-containing and SelD-like-lacking thermoproteales, it is possible to identify additional genes associated with SelD-like.

We analyzed the presence of all the genes annotated in the *P. arsenaticum* genome in other sequenced thermoproteales, and focused on those genes that occur exclusively in organisms containing SelD-like gene and are absent in organisms that lack this gene. Top candidate genes that co-occur with SelD-like are shown in Table 1, including SirA-like and Prx-like genes mentioned above.

**Table 1:** ***P. arsenaticum*** **genes that are present exclusively in all SelD-like-containing thermoproteales**

GI number	Gene annotation	Conserved domains (COG/Pfam/Others)	Occurrence in sulfolobales	Occurrence in other archaea and bacteria
145591425	Hypothetical protein Pars_1207	COG2210, peroxiredoxin family protein	+ (all)	+ (many archaea and bacteria)
145591427	SirA family protein	pfam01206, SirA-like protein	+ (all)	+ (many archaea and bacteria)
145591451	Adenylylsulfate reductase subunit beta	TIGR02060, adenosine phosphosulphate reductase, beta subunit;	-	+ (some archaea and many bacteria)
145591452	Adenylylsulfate reductase subunit alpha	TIGR02061, adenosine phosphosulphate reductase, alpha subunit	-	+ (some archaea and many bacteria)
145590950	Antibiotic resistance (efflux) protein	pfam07690, major facilitator superfamily	+ (all)	+ (many archaea and bacteria)

It is interesting that the adenylylsulfate reductase, or called adenosine 5′-phosphosulfate (APS) reductase, was found to be strongly related to SelD-like gene. This enzyme contains two subunits (AprA and AprB), which participates in S metabolism and catalyzes the reduction of APS to sulfite and AMP [37]. However, both genes were absent in sequenced sulfolobales genomes, implying the lack of such a relationship in this archaeal order that may use an unknown enzyme instead of AprA/B during S metabolism. Further analysis of the occurrence of AprA and AprB genes in other prokaryotes revealed that they are still highly conserved in many other sulfate-reducing and S-oxidizing prokaryotes (data not shown).

Another related gene is a predicted transporter that contains three transmembrane regions. It belongs to a large and diverse group of transporters (pfam07690, major facilitator superfamily), which includes uniporters, symporters, and antiporters of a variety of substrates such as ions, sugar phosphates, drugs, nucleosides, amino acids, and peptides. This gene is found in all sulfolobales and many other prokaryotes. However, its function is unclear. Further studies are needed to verify its biological role and the relationship with SelD-like gene.

### A possible model for the involvement of SelD-like protein in S metabolism in Crenarchaeota

**Figure 5:**
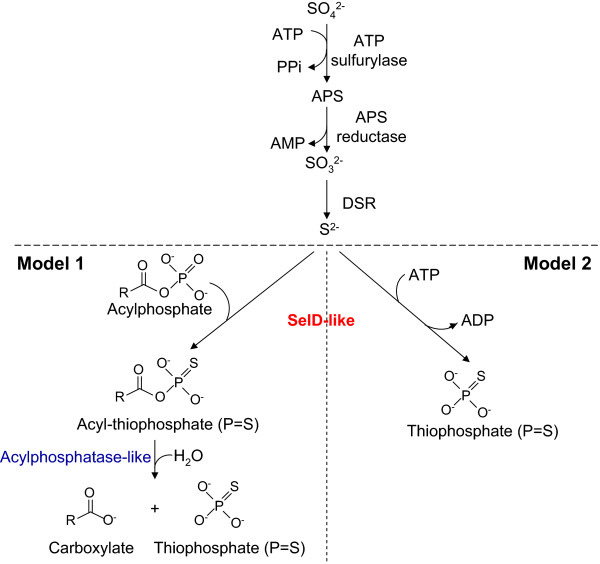
**A predicted model for the involvement of SelD-like protein in the S metabolism in thermoproteales.** Known S-related genes are shown in black, whereas SelD-like and acylphosphatase-like proteins are highlighted in red and blue, respectively.

It has been reported that many S-reducing archaea carries out sulfate reduction via the pathway originally proposed for bacterial species [38, 39]. After sulfate transport across the cytoplasmic membrane, it is first activated with ATP to form APS and pyrophosphate, which is catalyzed by ATP sulfurylase. Then APS reductase catalyzes the reduction of APS to sulfite and AMP. After that, the enzyme DSR catalyzes the six-electron reduction of sulfite to sulfide (Figure 5). The natural electron donors for APS reductase and DSR are not known.

Based on the results described above, the SelD-like protein may play a key role in the biosynthesis of thiophosphate (P=S) compound in sulfolobales and thermoproteales. In this study, two possible models were suggested (Figure 5). In Model 1, the substrates include sulfide and certain acylphosphate, and the product is considered as acyl-thiophosphate (P=S), which may be further hydrolyzed by the acylphosphatase-like protein (fused with SelD-like in thermoproteales). In Model 2 whose reaction is more similar to that for SelD, the substrates include sulfide and ATP, whereas the products are thiophosphate (P=S) and pyrophosphate (PPi); however, the functional link between SelD-like and acylphosphatase-like proteins is not clear in this model.

An interesting hypothesis is that SelD-like protein might be involved in cysteinyl-tRNA^Cys^ biosynthesis based on a pathway similar to Sec biosynthesis. It has been known that cysteinyl-tRNA^Cys^ is typically synthesized by cysteinyl-tRNA synthetase (CysS). In methanogenic archaea that lack the CysS gene, cysteinyl-tRNA^Cys^ is synthesized by O-phosphoseryl-tRNA synthetase (SepS) that acylates tRNA^Cys^ with phosphoserine (Sep), and Sep-tRNA: Cys-tRNA synthase (SepCysS) that converts Sep-tRNA^Cys^ to Cys-tRNA^Cys^ [40]. One possibility is that the thiophosphate (P=S) compound produced by SelD-like protein may be a special S donor for the tRNA-dependent Cys formation in these organisms. To test this idea, we analyzed the occurrence of CysS, SepS and SepCysS genes in SelD-like-containing crenarchaeota. All of them only have orthologous CysS genes. The lack of SepS and SepCysS genes in these organisms suggest that they do not have a known tRNA-dependent Cys formation pathway. It is possible that the special product of SelD-like protein is only present in sulfolobales and thermoproteales. However, additional genes and specific mechanisms related to S metabolism may have evolved in other S-reducing thermophiles. It is also possible that the occurrence of SelD-like gene in hyperthermophilic S-reducing archaea may just reflect a fundamental correlation, which is unrelated to S metabolism. In the future, experimental evidence is required to test these hypotheses.

## Conclusions

In this study, we carried out comparative and phylogenetic studies of SelD family in all sequenced archaea. Our data revealed restricted occurrence of SelD as well as limited utilization of Se in this kingdom. In addition, a distant homolog of SelD (SelD-like) was found to form a separate family in hyperthermophilic S-utilizing Crenarchaeota: Sulfolobales and Thermoproteales. The SelD-like protein has the same catalytic Cys as well as other functional residues as those in SelD proteins. Further sequence and genome context analysis of SelD-like genes revealed that they might be involved in S metabolism in these organisms. We also analyzed patterns of gene occurrence in different thermoproteales at a genome-wide level, and identified several genes that may co-occur with SelD-like gene. Finally, a model for the possible function of SelD-like protein in these organisms was proposed, which may provide insights into understanding the relationship between Se and S utilization in biology.

## Methods

### Genomic databases and sequences

Sequenced archaeal genomes from the Entrez Microbial Genome Database at NCBI were used in this study [41]. A total of 215 organisms were analyzed (as of August 2013). The *M. jannaschii* SelD protein (gi: 190352215) was used as query sequence to search for SelD homologs in archaea. TBLASTN program was used to identify genes coding for homologs with the cutoff of E-value ≤0.1. In addition, other key components of different Se utilization traits (such as archaeal SecS, SelB, YbbB, YqeB and YqeC) from representative organisms were also used for identification of the occurrence of each Se trait in archaeal species. Orthologous proteins were defined using the conserved domain database (COG, Pfam, TIGR, etc.) and bidirectional best hits [42].

### Multiple sequence alignment and phylogenetic tree reconstruction

Multiple sequence alignments were performed by Clustal Omega program (Version 1.2.0) [43] with number of iterations set to 2. The resulting multiple alignments were then checked for conservation of functional residues and manually edited. Phylogenetic trees were reconstructed by PHYLIP programs [44]. The PROML program was used for producing the phylogenetic trees by the maximum likelihood method with the Jones-Taylor-Thornton (JTT) probability model and the global rearrangements option was used. Bootstrap with 100 repetitions was carried out by SEQBOOT program to assess the confidence degree of nodes in the phylogenetic trees. CONSENSE program was used for computing the consensus tree. The neighbor-joining (NJ) method was used for the phylogenetic reconstruction to validate the tree topology with NEIGHBOR program. Robustness of these phylogenies was evaluated by two additional programs, PHYML (maximum likelihood analysis) [45] and MrBayes (Bayesian estimation of phylogeny) [46] with different parameters (for example, transition matrices and evolutionary models).

### Identification of genes associated with SelD-like gene

Initial searches for the occurrence of SelD-like genes revealed that only the order Thermoproteales (in the archaeal phylum Crenarchaeota) has both SelD-like-containing and SelD-like-lacking organisms (9 and 3 organisms, respectively). To identify genes associated with SelD-like, we adopted a modified strategy which had been used previously to identify the archaeal/eukaryotic SecS gene [47]. Protein sequences of all annotated genes in *P. arsenaticum* (a representative thermoproteales containing SelD-like gene) were used as a query dataset. BLAST programs were used to search these sequences against all sequenced thermoproteales genomes using the following criteria: (i) e-value is less than 1e-07; (ii) the length of the alignment should be extending over half the length of the query sequence; and (iii) the alignment identity should be at least 30%. A simple Perl script was developed to parse the BLAST output and examine presence/absence of homologs in all analyzed genomes. Genes present in any of the thermoproteales lacking SelD-like were dismissed. The remaining genes were then searched against thermoproteales containing SelD-like to determine their occurrences. Top candidate genes were further analyzed for possible function. The occurrence of these candidates in SelD-like-containing sulfolobales was also analyzed.

### Availability of supporting data

The data sets supporting the results of this article are included within the article and its additional files.

## Electronic supplementary material

Additional file 1: Table S1: This file contains Table S1, which shows the distribution of SelD, SelD-like and all known Se utilization traits in archaea. (XLS 48 KB)

Additional file 2: Figure S1: This file contains two supplementary figures: **Figure S1.** shows the multiple sequence alignment of SelD and SelD-like proteins. **Figure S2.** shows genomic context of SelD-like gene in completely sequenced genomes that contain this gene. (DOC 178 KB)

Additional file 3: **This file contains representative SelD and SelD-like protein sequences used for multiple sequence alignment (Figure** 2 **and Additional file** 2 **: Figure S1).** (TXT 6 KB)

## Abbreviations

Se: Selenium
Sec: Selenocysteine
Cys: Cysteine
SelA: Sec synthase
SelB: Sec-specific elongation factor
SelC: Sec-tRNA^[Ser]Sec^
SelD: Selenophosphate synthetase
PSTK: O-phosphoseryl-tRNA^[Ser]Sec^ kinase
SecS: Archaeal/eukaryotic Sec synthase
SBP2: SECIS-binding protein 2
SeU: 5-methylaminomethyl-2-selenouridine
PurM: Phosphoribosylaminoimidazole synthetase
PurL: Formylglycinamide ribonucleotide amidotransferase
ThiL: Thiamine-monophosphate kinase
HypE: Nickel-iron hydrogenase maturation protein
ATP: Adenosine 5′-triphosphate
S: Sulfur
SCL: Sec lyase
CD: Cys desulfurases
Asp: Aspartic acid
Asn: Asparagine
Prx: Peroxiredoxin
DSR: Dissimilatory sulfite reductase
APS: Adenosine 5′-phosphosulfate
PPi: Pyrophosphate
CysS: Cysteinyl-tRNA synthetase
SepS: O-phosphoseryl-tRNA synthetase
Sep: Phosphoserine
SepCysS: Sep-tRNA:Cys-tRNA synthase.
