# Supplementary material for: Computational identification of a new SelD-like family that may participate in sulfur metabolism in hyperthermophilic sulfur-reducing archaea
Source: BMC Genomics. 2014 Oct 17;15(1):908. doi: 10.1186/1471-2164-15-908 (PMC4210487; doi:10.1186/1471-2164-15-908)
Supplement: Supplementary file 2 — Additional file 2: Figure S1: This file contains two supplementary figures: Figure S1. shows the multiple sequence alignment of SelD and SelD-like proteins. Figure S2. shows genomic context of SelD-like gene in completely sequenced genomes that contain this gene. (DOC 178 KB) [file 12864_2013_6599_MOESM2_ESM.doc]

**Supplemental figures**

**Figure S1. Multiple sequence alignments of SelD and SelD-like proteins.** Conserved residues are highlighted. Sec (U) and the corresponding Cys (C) residues are shown in red and blue, respectively. Several other residues that are experimentally verified to be essential for catalysis in SelD (including Asp, Lys and Asn residues) are highlighted in purple.

**Figure S2. Genomic context of SelD-like gene in all completely sequenced genomes that contain SelD-like gene.** Functionally related genes, including the acylphosphatase-like, SirA-like, Prx-like proteins, are color coded as noted in Fig. 4. Coding direction is also indicated.

**Figure S1**

**SelD**

Haloarcula_hispanica 1 --------MGTDTAADGDEETTRLTEYTELHG**C**SC**K**VGQSDLDSLLADAG-LTGD--SDA
Methanoplanus_petrolearius 1 ---------------MSDIEKRKLTSIAPLFG**C**SC**K**LPETELETLLEDIN-I----SYSP
Methanocaldococcus_jannaschii 1 --------------MERGNEKIKLTELVKLHG**U**AC**K**LPSTELEFLVKGIV-TDDDLLDKN
Methanopyrus_kandleri 1 -----------------MSRKKSLVEMADLHG**U**AC**K**LPQGDLEDLLKGVE-LPEE--GGR
Escherichia_coli 1 ----------------MSENSIRLTQYSHGAG**C**GC**K**ISPKVLETILHSEQ-AKFVDPNLL
Syntrophomonas_wolfei 1 ------------------MKQTRLTEMVRAAG**U**AA**K**IGPEALESILKDFP-L-PVHPDLL
Ralstonia_eutropha 1 -----------------MAESIRLTQYSHGAG**C**GC**K**ISPKVLDVILAGSG-AQNLDPRLW
**SelD-like**

Acidianus_hospitalis 1 ----MEEVFERFRKNLEVYNKMGLNPLSLATG**C**AV**K**VDLID--TVYPALEKIKNKLE--N
Metallosphaera_cuprina 1 --MAMSDLFERFRDNLNKYKKMGINPLSLATG**C**AV**K**VDLID--TVYPALEKLKEKLLANN
Sulfolobus_tokodaii 1 -MHSNMDIFTKFKENLEVYKSMGLNPLSLATG**C**AV**K**VDLID--TVYPALEKIKKILEERN
Caldivirga_maquilingensis 1 MKVSKEERMSRFLKHVSKYASIGVNLTSLALG**C**SV**K**VDLYN--VLYPALSIVKRELGELH
Pyrobaculum_aerophilum 1 -----MNRLERFKERVKLYREAGIALESLSLG**C**SV**K**VDLYN--VLYPALQLLREEMYKLN
Thermoproteus_uzoniensis 1 -----MNPVEKFRERVRLYREAGIALESLSLG**C**SV**K**VDLYD--VLYPALELLRDDVRRLN
Vulcanisaeta_distributa 1 -MEMRDDRIRVFRERLDKYLDLGINLLSLAIG**C**SV**K**VDLYD--TLYPALSLVNNEIAKLN


**SelD**

Haloarcula_hispanica 50 LLFGVGE**D**AAARKLT---------------------------------------------
Methanoplanus_petrolearius 41 EILCPGD**D**AAVIKIN---------------------------------------------
Methanocaldococcus_jannaschii 46 ILVGLGD**D**ASIIKR----------------------------------------------
Methanopyrus_kandleri 41 VEVGVGD**D**AAVIRV----------------------------------------------
Escherichia_coli 44 VGNETRD**D**AAVYDLG---------------------------------------------
Syntrophomonas_wolfei 41 IGIESRD**D**AAVLKLD---------------------------------------------
Ralstonia_eutropha 43 VGNASRD**D**AAVYAIDGDD------------------------------------------
**SelD-like**

Acidianus_hospitalis 53 IEILPRE**D**ADIFITREKMEIKRIIGG-----------GNFDADRGVSLIQVNQDTAGNPE
Metallosphaera_cuprina 57 IEVMPRE**D**TDIFVSRESEVLKRVING-----------GEFDADRAISLIQVNQETAGNPE
Sulfolobus_tokodaii 58 ITILPRE**D**ADIFVSREKMEMKRLING-----------GEFDADRAITLIQVNQETAGNPD
Caldivirga_maquilingensis 59 IKIAPRE**D**VAVLRGS-ELDLMRVITSVDSPKLPINTISDFNPDALILLVESFQGNASDPD
Pyrobaculum_aerophilum 54 LVIAPRE**D**AAIMPGA-SAALRRYFLDVENPRLDPAEVEKLSPTVAIVLAQVYMGKAAAPD
Thermoproteus_uzoniensis 54 LVIAPRE**D**AAIMRGA-GAELRRLYLDPEDPHIDPAFLESYAPDLAVVLVQLYMAKAATPS
Vulcanisaeta_distributa 58 IEIQPRE**D**VAVLRSNGDYSLVRRIYDITGNGVNKDELVSINPSVALLLLQVHQSRASSPR


**SelD**

Haloarcula_hispanica 65 -----------------------DELALVST--------V**D**FF-----------------
Methanoplanus_petrolearius 56 -----------------------DDLAIIKT--------T**D**FF-----------------
Methanocaldococcus_jannaschii 60 -----------------------NGLVIAKT--------V**D**VF-----------------
Methanopyrus_kandleri 55 -----------------------DGGYVIQS--------V**D**FF-----------------
Escherichia_coli 59 -----------------------NGTSVIST--------T**D**FF-----------------
Syntrophomonas_wolfei 56 -----------------------EEKALIQT--------I**D**FF-----------------
Ralstonia_eutropha 61 -----------------------SGRGVVST--------T**D**FF-----------------
**SelD-like**

Acidianus_hospitalis 102 KFANFLLKTYSSIRS-RRKLTIGKGHSIVTTIPSAEVAVL**D**LIKLDGSKINSYTLANNDT
Metallosphaera_cuprina 106 KFANFLLKIYTGVKT-RRKLIVGKGHSIVTTNPKAEVGIL**D**LFKLDGRELRSYTLSNNDT
Sulfolobus_tokodaii 107 AFSEFLVRVYTSIKT-SRKLTIGKGHSIVTTKKDGEVAVL**D**LFRLEGREEKSYTVANNDT
Caldivirga_maquilingensis 118 SFANIMIRLFKELAKVNKELVIGKGHSIVSTQPNASIHVL**D**FIKVKNK-DDAYTLVNNDT
Pyrobaculum_aerophilum 113 LFAKYVAGLYKALGSSRHKVWLGKGHSIISTKKGAEFFMV**D**FLKAEG--QEGYIVANNDT
Thermoproteus_uzoniensis 113 KFAEYAARLYKALGSSRHRVWLGKGHSIVSTKKGAEFFMV**D**FLKAEP--GEGYVLANNDT
Vulcanisaeta_distributa 118 EFASSIISLYRRLGSSPVRVRIGKGHSIVSTKEKAEFALI**D**FISTKS--GDGYLLANNDT


**SelD**

Haloarcula_hispanica 77 ----TPIV-DDPYDFGRVAAC**N**AAS**D**AFATGAVENVDCLVVLGLPRE-LTD-SAASILAG
Methanoplanus_petrolearius 68 ----TPIV-DDPYIQGKIAAC**N**ATN**D**VYAMGATEIVGVLALLGIPRE-LPLENARMMLKG
Methanocaldococcus_jannaschii 72 ----TPIV-DDPYIQGKIAAC**N**STS**D**IYAMGLLDIVGVLAIVGIPEK-LPIHVVREMLKG
Methanopyrus_kandleri 67 ----TPIH-PDPYTQGRIAAN**N**SIN**D**VFAMGATEVLSVLVVSGFPRE-LPEEDAREMLQG
Escherichia_coli 71 ----MPIV-DNPFDFGRIAAT**N**AIS**D**IFAMGGKPIMA-IAILGWPINKLSPEIAREVTEG
Syntrophomonas_wolfei 68 ----TPMV-DDPFIFGQIAAT**N**ALN**D**IYAMGGTPILA-LNVVCFPEC-ADLQVLRKILEG
Ralstonia_eutropha 73 ----MPIV-DDPFDFGRIAAT**N**AIS**D**IYAMGGDPLMA-IAILGWPVNVLPPEVAREVVAG
**SelD-like**

Acidianus_hospitalis 161 IQIVDPLEDPGSQMQVDVAIS**N**SLN**D**LFTKGAYQNLKMLPLFDAPND----DLKERIMKN
Metallosphaera_cuprina 165 IQIVDPLDDPGSQMQVDVGVS**N**SLN**D**LFTKGAFQDLKMIPVADAPDP----DLKKTLLNN
Sulfolobus_tokodaii 166 IQIVDPLDNPGSQVQVDVAIS**N**SLN**D**LFTKGVFQELTVVPVVDAPNS----ELKKKLLSN
Caldivirga_maquilingensis 177 IQVIDPMDEIASRRQVSVALN**N**ALN**D**LFSKGAFKDLEFYPVYDAPGE----Y-KHGLFNE
Pyrobaculum_aerophilum 171 IQVIDPSEDFDSPLQIAVAVN**N**ALN**D**LFTKGAWKDIHIAPVYDAPPP----F-RGPLEAR
Thermoproteus_uzoniensis 171 IQVIDPSEDFDSPLQAAVAVN**N**ALN**D**LYVKGVYKDVEIAPVYDAPEP----Y-RARVKAA
Vulcanisaeta_distributa 176 IQIIDPTEDPGSYRQVATAVS**N**ALN**D**LFIKGVYRDITIYPVYDAPIE----DLKEKLMKN

**SelD**

Haloarcula_hispanica 130 MADALDAMDGVI-AGGHTIMSPWPFAGGAISATARPDALLTSQGASPGDRLYLTKPLGTQ
Methanoplanus_petrolearius 122 FQDFCNSIGTSI-VGGHTIINPWPFIGGAVTAISSPDKIVYHSGAQPGDVLLLTKPLGIQ
Methanocaldococcus_jannaschii 126 FQDFCRENKTTI-VGGHTILNPWPLIGGAVTGVGREEEVLTKAGVKVGDVLILTKPLGTQ
Methanopyrus_kandleri 121 FADQCREVDALI-VGGHTIMNPWPILGGCVTGFA--ERYVTVGGAEPGDVLYLTKPLGTQ
Escherichia_coli 125 GRYACRQAGIAL-AGGHSIDAPEPIFGLAVTGIVPTERVKKNSTAQAGCKLFLTKPLGIG
Syntrophomonas_wolfei 121 GLSKVLEAGALL-VGGHTVDDNEPKYGLAVSGLVHPQKIIANNGAQPGDLLFLTKPLGNG
Ralstonia_eutropha 127 GRKACDDAGIPL-AGGHSIDAPEPIFGLAVTGMVDRMHMKRNDTATAGCRLYLTKPIGIG
**SelD-like**

Acidianus_hospitalis 217 YENYSEKYDIPI-INDIQPNTKSLMLGATVLGESDHELPTYYDKVEEGDEIIVTRPIGEL
Metallosphaera_cuprina 221 FERFSKRYNVEL-LTDVQPSTGTLMIGATVVGKSDHELPTYYDKVDEGMEILVTRPVGEL
Sulfolobus_tokodaii 222 YENYVKKYNMNL-RNDIQPSVGTLMMGATVVGKSDHELPTFYDKVNENMVIITTRFFGEL
Caldivirga_maquilingensis 232 VKAYVNELGGRL-HDVEQPNLGYLLIGSTVASRLDREPPMFYNEVKEGFKIIVTRPFGEL
Pyrobaculum_aerophilum 226 VKSYASSLGKL--VEAPQPEMGYLLLGATAYARLDREPPLFYDKIREGFVVVVTRPFGEL
Thermoproteus_uzoniensis 226 VESHAASLGRL--VEAPQPGRGYLLLGATAYGRLDREPPTYYSQLGEGFVVLVTRPFGEL
Vulcanisaeta_distributa 232 FREFSSKWDLQLSTDVQQPRVNYLLMGATVVGTLDREPPMFYDNIKAGFKILITRPFGEL


**SelD**

Haloarcula_hispanica 189 ----PAMGATRVTDAQFVETVTDAADRPLDSIASEAIAWMTTPNRDATLACR--------
Methanoplanus_petrolearius 181 ----PAMASTRLSSEYSSIIQSTLDSDIVANAVNLAIECMTTSNLNAAKAIN--------
Methanocaldococcus_jannaschii 185 ----TAMALSRIPEEFKDLISIT--EEERDYIINKAIEIMTTSNRYALKALRKAEERVG-
Methanopyrus_kandleri 178 ----PAMAALRLPEDVRKQFLT---DSELEEAVDLAVEVMTEPLKDAAEAAL--------
Escherichia_coli 184 ----VLTTAEKK-------------SLLKPEHQGLATEVMCRMNIAGASFAN--------
Syntrophomonas_wolfei 180 ----VIATSIKA-------------EMVSGEAYKEAIKWMSMLNRESSQAMM--------
Ralstonia_eutropha 186 ----VLTTAEKK-------------GLLRPEHAHVARDWMCVLNRPGSAFGR--------
**SelD-like**

Acidianus_hospitalis 276 APINVYLWILVVPEVLELMESRGISFKRLEKVKMEVIEQMRKPNIPTAKVIYNYLPEFGK
Metallosphaera_cuprina 280 TPINVHMWLLTVPELIDTMEERGITLNKVEEAKKRALDYMTKPNFDSAEVIYNHLPEFGG
Sulfolobus_tokodaii 281 TPINVHLWSLAVPELIEMMESRGISFSRIEKVKSEALEIMRRPNIHVAKVIYSYLPEFGK
Caldivirga_maquilingensis 291 SIIGTYVALHVDEELYDKFEREVMTVDELEEIKEYALNQMAKPNIDVAKVIYNHLPELGR
Pyrobaculum_aerophilum 284 AYFTTYVAVHTDETLMKKFEEEVMPIEQFEAEKRRVLEIMATPNLEAAKAIYQYLPDLGE
Thermoproteus_uzoniensis 284 AYFTTYVAVNTDEELLKAFEKSVMPLDQFEKEKRRVLELMAAPNADVARVIYDHLPDLGE
Vulcanisaeta_distributa 292 SIISTYLTAHVDESIMDELERNVMSIEDLEKLKIKIMDILSRPNIEIARVISKYLPEIGE


**SelD**

Haloarcula_hispanica 237 -----EYATAAT**D**ITGFGLAGQSRVMADRSNVGIEL--THLPVIAGTPALSTLFG-----
Methanoplanus_petrolearius 229 ----EVGANALT**D**VTGFGLYGHGLNIARKSNVSLNI--DLIPIISGTLELSALFE-----
Methanocaldococcus_jannaschii 238 ----DKIANALT**D**ITGFGILGHSNEMAKNSNVLIEI--NLLPCIKRTPELSRLFG-----
Methanopyrus_kandleri 223 ----EVGVHAMT**D**VTGFGLKGHAGEMAEASGVRVVI--ERLPVIPGTTELSRALG-----
Escherichia_coli 219 ----IEGVKAMT**D**VTGFGLLGHLSEMCQGAGVQARVDYEAIPKLPGVEEYIKLGAVPGGT
Syntrophomonas_wolfei 215 ----EVGVNAAT**D**ITGFGLMGHLYEMAWGSDVQVEVFADKVPFMEGTLEYAGLGLIPGGA
Ralstonia_eutropha 221 ----LAGVRAMT**D**VTGFGLLGHLVEMADGSGLTARLDYAAVPVLPEIRRYIDAGCVPGGT
**SelD-like**

Acidianus_hospitalis 336 SFDRENYITMTT**D**VTGPGIFV-IKEFAEKAGVDVRL--DKIPVIDEEIAEFATEG-----
Metallosphaera_cuprina 340 KFDLESHVAMTT**D**VTGPGIFV-VKEFAEKALVDVDL--YDVPVIDREISEFATEN-----
Sulfolobus_tokodaii 341 SFDEKSHIAMTT**D**VTGPGIFV-IKEFAEKAKVDVEL--NDIPVIDRDICEFATEN-----
Caldivirga_maquilingensis 351 RFNDDEHVAATI**D**ISGPGIFV-FKELAETANVKIRL--SNIPLISPEVAEFAASN-----
Pyrobaculum_aerophilum 344 RFDPEAHIAATI**D**VSGPGIFV-FKEVAERAGVDIRL--FDVPLMSSAVSKFAADN-----
Thermoproteus_uzoniensis 344 RFDPEAHIAATI**D**ISGPGVFV-FKEVAERAGVDVEL--WDVPLLGPSVSRFAAEN-----
Vulcanisaeta_distributa 352 AFREDEHIAATI**D**VSGPGIFV-FKELAEQANVDVAL--YNIPLISPEVARFAAEH-----


**SelD**

Haloarcula_hispanica 285 ------YG-------------LTDGESAETSGGLFVSVPPAATDAVEAAFDD-ASVFYRA
Methanoplanus_petrolearius 278 ------HG-------------LEEGKSAETAGGLLMSVSEENLDEIIHSLNK-YNVPAYE
Methanocaldococcus_jannaschii 287 ------HA-------------LLDGYGAETAGGLLISAKEEYKDNLIDELEK-AKCYAFE
Methanopyrus_kandleri 272 ------YG-------------LERGESAETAGGLLVAVPEEHAEDLEDAFER-RDVWYRR
Escherichia_coli 275 ERNFASYG---HLMGEMPREVRDLLCDPQTSGGLLLAVMPEAENEVKATAAE-FGIELTA
Syntrophomonas_wolfei 271 YNNRDYLKDKVEYAGNIDPLIRDLFFSPETAGGLLIAVAEKKAGELLQVMEK-RGSFCNL
Ralstonia_eutropha 277 QRNFDSYG---HRIGTLTDEQRALLCDPQTSGGLLVAVEPAGEAAFLDACTR-LGLDLAP
**SelD-like**

Acidianus_hospitalis 388 ------FI--------------IPNSTSGTNGATVVFYSRRISDDIIEDLQK-EGLKPIK
Metallosphaera_cuprina 392 ------FI--------------IPNSTAGTNGAIVIFASKKVIDDIAQDLTK-RGLEPRI
Sulfolobus_tokodaii 393 ------FI--------------IPNSTIGTNGAIVMFADKRIADDIMEDLIK-VGEKPEI
Caldivirga_maquilingensis 403 ------YI--------------ISDATAGTNGAVALVVSSGLVDDVLDELGKIPGLKPMV
Pyrobaculum_aerophilum 396 ------YI--------------MPDATAGTNGAIAIFASRKVAEELVEKLSKAPHAKPTV
Thermoproteus_uzoniensis 396 ------FI--------------MPDATAGTNGAIAVFLHKKLADEVLDELSKIPRLRPAV
Vulcanisaeta_distributa 404 ------YI--------------ITDATAGTNGAIAIVASKDVIDSIVRDLRGIEDVQPMV

**SelD**

Haloarcula_hispanica 325 VGRVTGGSGVTLDDLTLEEVRS--------------------------------------
Methanoplanus_petrolearius 318 IGTVKEKGNEHVVIDNPEILEISSLSVKFES-----------------------------
Methanocaldococcus_jannaschii 327 VGRVVKKGEGKAVLSKDVKVIEI-------------------------------------
Methanopyrus_kandleri 312 IGRVEEGSGVEVRGDVEEV--EDYP-----------------------------------
Escherichia_coli 331 IGELVPARGGRAMVEIR-------------------------------------------
Syntrophomonas_wolfei 330 IGRVRGEHFSPIRVRDSRGAEGQNP-----------------------------------
Ralstonia_eutropha 333 IGELAARGEHAVEVA---------------------------------------------
**SelD-like**

Acidianus_hospitalis 427 IGKVLGKGQGVVHVNKDIERFIHRKNVLSHFKID--------------------------
Metallosphaera_cuprina 431 IGRVLRKGTGVVYVKKDVCKLIHRENILKHFKVRDAN-----------------------
Sulfolobus_tokodaii 432 VGYVKGKGNGTVYAPKDVMKLIRRDNVLKQFKIKE-------------------------
Caldivirga_maquilingensis 443 IGEVVGRGEGTLIVPDYVTRYIKDKVMLTKLTIASNILQGVSRQ-AALRLARAEIMVTGK
Pyrobaculum_aerophilum 436 IGVVEGKGEGRLIVPEWALQYISSKKLREKLG-AASVLGGLARVVG--RPIRAVAYVEGA
Thermoproteus_uzoniensis 436 VGRVLGKGEGRLAVPREALAYISSEKLREKLVGAAQVLGGLA---G--KAVRARAYLEGD
Vulcanisaeta_distributa 444 IGEVMGKGSGRLFVPDYVTRYITSKSLLMKLTMNIDVLRSLRRQQVKCEKVRVEARVFGN


**SelD**

Haloarcula_hispanica ------------------------------------------------------------
Methanoplanus_petrolearius ------------------------------------------------------------
Methanocaldococcus_jannaschii ------------------------------------------------------------
Methanopyrus_kandleri ------------------------------------------------------------
Escherichia_coli ------------------------------------------------------------
Syntrophomonas_wolfei ------------------------------------------------------------
Ralstonia_eutropha ------------------------------------------------------------
**SelD-like**

Acidianus_hospitalis ------------------------------------------------------------
Metallosphaera_cuprina ------------------------------------------------------------
Sulfolobus_tokodaii ------------------------------------------------------------
Caldivirga_maquilingensis 502 VQGVGFRPLVRRNAKSLGLTGFARNNEDGSVLIIVEGEEGNIKAFIESLRNINIAEVREL
Pyrobaculum_aerophilum 493 VQGVGFRPMARARAKALGLLGYAKNLPDGRVEVVVEGDEERVRKYVEELCK-GFENCRVG
Thermoproteus_uzoniensis 491 VQGIGFRPTARAKARALGLTGYAANLPDGRVELVVEGDRDRVEKLLQELCA-RF-NCRVA
Vulcanisaeta_distributa 504 VQGVGFRPTLRRQALSLGLTGYVRNLPDGSVEVVAEGCKEDVMALIEWIRSSPVGSVETI


**SelD**

Haloarcula_hispanica -----------------
Methanoplanus_petrolearius -----------------
Methanocaldococcus_jannaschii -----------------
Methanopyrus_kandleri -----------------
Escherichia_coli -----------------
Syntrophomonas_wolfei -----------------
Ralstonia_eutropha -----------------
**SelD-like**

Acidianus_hospitalis -----------------
Metallosphaera_cuprina -----------------
Sulfolobus_tokodaii -----------------
Caldivirga_maquilingensis 562 NIKWSSYKGEFQDFTIE
Pyrobaculum_aerophilum 552 QVIYAEARGEFSDFSIL
Thermoproteus_uzoniensis 549 ELAWEPAEGAYKDFEIR
Vulcanisaeta_distributa 564 NYVIKQYFGEFEDFEIR

**Figure S2**

**Crenarchaeota/Sulfolobales**

***Acidianus hospitalis***

**SelD-like**

COG0675, Transposase and inactivated derivatives

COG3384, Aromatic ring-opening dioxygenase, catalytic LigB subunit related

COG2210, Peroxiredoxin family protein

pfam06525, Sulfocyanin (SoxE)

pfam00355, Rieske [2Fe-2S] domain

pfam06525, Sulfocyanin (SoxE)

Quinol oxidase subunit A and B

pfam07185, Protein DUF1404

COG1529, Aerobic-type carbon monoxide dehydrogenase, large subunit

HP

HP

cd02756, Arsenite oxidase

***Metallosphaera cuprina Ar-4***

**SelD-like**

cd06223, Phosphoribosyl transferase (PRT)-type I domain

pfam03464, eRF1 domain 1/2

pfam01925,

Sulfite exporter TauE/SafE

COG1529, Aerobic-type carbon monoxide dehydrogenase, large subunit

pfam09969, protein DUF2203

COG1331, Highly conserved protein containing a thioredoxin domain

***Metallosphaera sedula***

**SelD-like**

COG0607, Rhodanese-related sulfurtransferase

COG2107, Predicted periplasmic solute-binding protein

COG1986, Inosine/xanthosine triphosphatase

HP

COG1529, Aerobic-type carbon monoxide dehydrogenase, large subunit

pfam09969, protein DUF2203

HP

HP

pfam00085, Thioredoxin

COG0665, Glycine/D-amino acid oxidases

pfam04414, D-aminoacyl-tRNA deacylase

HP

COG1331, Highly conserved protein containing a thioredoxin domain

3-ketoacyl-(acyl-carrier-protein) reductase

COG0463, Glycosyltransferases involved in cell wall biogenesis

COG3371,

Predicted membrane protein

pfam14010, Phosphoenolpyruvate carboxylase

HP

***Sulfolobus acidocaldarius N8***

**SelD-like**

COG0607, Rhodanese-related sulfurtransferase

HP

pfam07185, Protein DUF1404

Aerobic-type carbon monoxide dehydrogenase, medium, small and large subunits

COG1592, Rubrerythrin

COG0247, Fe-S oxidoreductase

HP

Quinol oxidase polypeptide I/III

pfam06525, Sulfocyanin (SoxE)

pfam00355, Rieske [2Fe-2S] domain

pfam12007, Protein DUF3501

***Sulfolobus islandicus HVE10/4***

**SelD-like**

COG1252, NADH dehydrogenase, FAD-containing subunit

pfam09941, protein DUF2173

COG2044, Predicted peroxiredoxins

Aerobic-type carbon monoxide dehydrogenase, medium, small and large subunits

HP

COG1592, Rubrerythrin

COG0247, Fe-S oxidoreductase

pfam12007, Protein DUF3501

COG0607, Rhodanese-related sulfurtransferase

COG3388, Predicted transcriptional regulator

cd09898, H3TH domain

pfam07849, Protein DUF1641

COG2210, Peroxiredoxin family protein

pfam01206, SirA-like protein

***Sulfolobus solfataricus 98/2***

**SelD-like**

COG1319/COG2080/COG1529,

Aerobic-type carbon monoxide dehydrogenase , middle/small/large subunit

HP

COG0842,

ABC-type multidrug transport system, permease componen

COG1131,

ABC-type multidrug transport system, ATPase component

HP

COG0446, Uncharacterized NAD(FAD)-dependent dehydrogenases

Phenylacetate-CoA ligase

COG1545,

Predicted nucleic-acid-binding protein containing a Zn-ribbon

acetyl-CoA acetyltransferase

***Sulfolobus tokodaii str. 7***

**SelD-like**

pfam07849, Protein DUF1641

COG2210, Peroxiredoxin family protein

COG0446, Uncharacterized NAD(FAD)-dependent dehydrogenases

COG2084, 3-hydroxyisobutyrate dehydrogenase and related beta-hydroxyacid dehydrogenases

pfam09969, protein DUF2203

COG0308, Aminopeptidase N

pfam00115, Cytochrome C and Quinol oxidase polypeptide I

HP

COG0607, Rhodanese-related sulfurtransferase

HP

HP

HP

HP

**Crenarchaeota/Thermoproteales**

***Caldivirga maquilingensis IC-167***

**SelD-like**

COG1716, FOG: FHA domain

COG0446, Uncharacterized NAD(FAD)-dependent dehydrogenases

COG0515, Serine/threonine protein kinase

COG1793, ATP-dependent DNA ligase

COG0438, Glycosyltransferase

COG0181, Porphobilinogen deaminase

pfam01206, SirA-like protein

HP

COG2017, Galactose mutarotase and related enzymes

pfam04894, Archaeal protein DUF650

HP

***Pyrobaculum aerophilum str. IM2***

**SelD-like**

COG2046, ATP sulfurylase (sulfate adenylyltransferase)

COG2181, Nitrate reductase gamma subunit

COG2221, Dissimilatory sulfite reductase (desulfoviridin), alpha and beta subunits

pfam05168, HEPN domain

HP

pfam01206, SirA-like protein

pfam01206, SirA-like protein

HP

HP

HP

COG2210, Peroxiredoxin family protein

HP

HP

***Pyrobaculum arsenaticum DSM 13514***

**SelD-like**

COG2221, Dissimilatory sulfite reductase (desulfoviridin), alpha and beta subunits

COG1587, Uroporphyrinogen-III synthase

COG1331, Highly conserved protein containing a thioredoxin domain

HP

COG3350, Uncharacterized conserved protein

COG2210, Peroxiredoxin family protein

COG1249, Pyruvate/2-oxoglutarate dehydrogenase complex, dihydrolipoamide dehydrogenase (E3)

COG1225, Peroxiredoxin

pfam01206, SirA-like protein

pfam01206, SirA-like protein

COG2524, Predicted transcriptional regulator

***Pyrobaculum calidifontis JCM 11548***

**SelD-like**

HP

HP

pfam05942, Archaeal PaREP1/PaREP8 family

HP

pfam01206, SirA-like protein

pfam01206, SirA-like protein

COG1587, Uroporphyrinogen-III synthase

COG1249, Pyruvate/2-oxoglutarate dehydrogenase complex, dihydrolipoamide dehydrogenase (E3)

COG1587, Uroporphyrinogen-III synthase

pfam00355, Rieske [2Fe-2S] domain

COG1741], Pirin-related protein

COG3391, Uncharacterized conserved protein

HP

***Pyrobaculum oguniense TE7***

**SelD-like**

COG2221, Dissimilatory sulfite reductase (desulfoviridin), alpha and beta subunits

HP

pfam01206, SirA-like protein

pfam01206, SirA-like protein

HP

COG2210, Peroxiredoxin family protein

COG1587, Uroporphyrinogen-III synthase

COG3350, Uncharacterized conserved protein

COG1331, Highly conserved protein containing a thioredoxin domain

COG1225, Peroxiredoxin

pfam02579], Dinitrogenase iron-molybdenum cofactor;

***Pyrobaculum sp. 1860***

**SelD-like**

pfam01978, Sugar-specific transcriptional regulator TrmB

HP

pfam01206, SirA-like protein

pfam01206, SirA-like protein

HP

COG2210, Peroxiredoxin family protein

COG2221, Dissimilatory sulfite reductase (desulfoviridin), alpha and beta subunits

COG3350, Uncharacterized conserved protein

COG1331, Highly conserved protein containing a thioredoxin domain

COG1225, Peroxiredoxin

HP

COG1587, Uroporphyrinogen-III synthase

***Thermoproteus uzoniensis 768-20***

**SelD-like**

pfam09376, NurA domain

COG1012, NAD-dependent aldehyde dehydrogenases

HP

pfam01206, SirA-like protein

pfam05942, Archaeal PaREP1/PaREP8 family

HP

pfam13472, GDSL-like Lipase/Acylhydrolase family

COG1192, ATPases involved in chromosome partitioning

pfam07788, Protein DUF1626

pfam06745, KaiC

HP

***Vulcanisaeta distributa DSM 14429***

**SelD-like**

HP

pfam01206, SirA-like protein

COG2210, Peroxiredoxin family protein

pfam01022, Bacterial regulatory protein, arsR family

COG1032, Fe-S oxidoreductase

COG0101, Pseudouridylate synthase

TIGR03154, cytochrome b558/566, subunit A

HP

pfam00355, Rieske [2Fe-2S] domain

COG1290, Cytochrome b subunit of the bc complex

pfam01206, SirA-like protein

HP

COG1976, Translation initiation factor 6 (eIF-6)

***Vulcanisaeta moutnovskia 768-28***

**SelD-like**

pfam01206, SirA-like protein

COG2210, Peroxiredoxin family protein

COG0656, Aldo/keto reductases, related to diketogulonate reductase

COG1032, Fe-S oxidoreductase

COG0101, Pseudouridylate synthase

COG1537, Predicted RNA-binding proteins

COG1645, Uncharacterized Zn-finger containing protein

pfam00083, Sugar (and other) transporter

COG0524, Sugar kinases, ribokinase family

pfam01206, SirA-like protein

HP

HP
